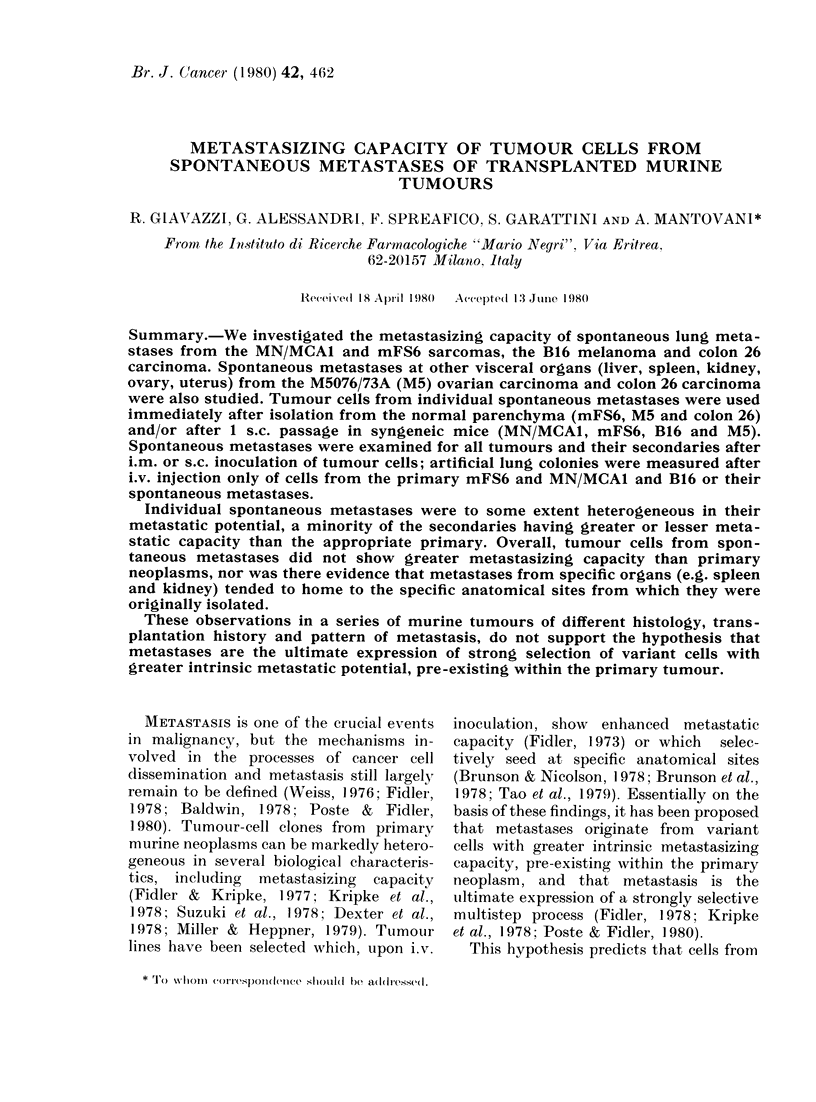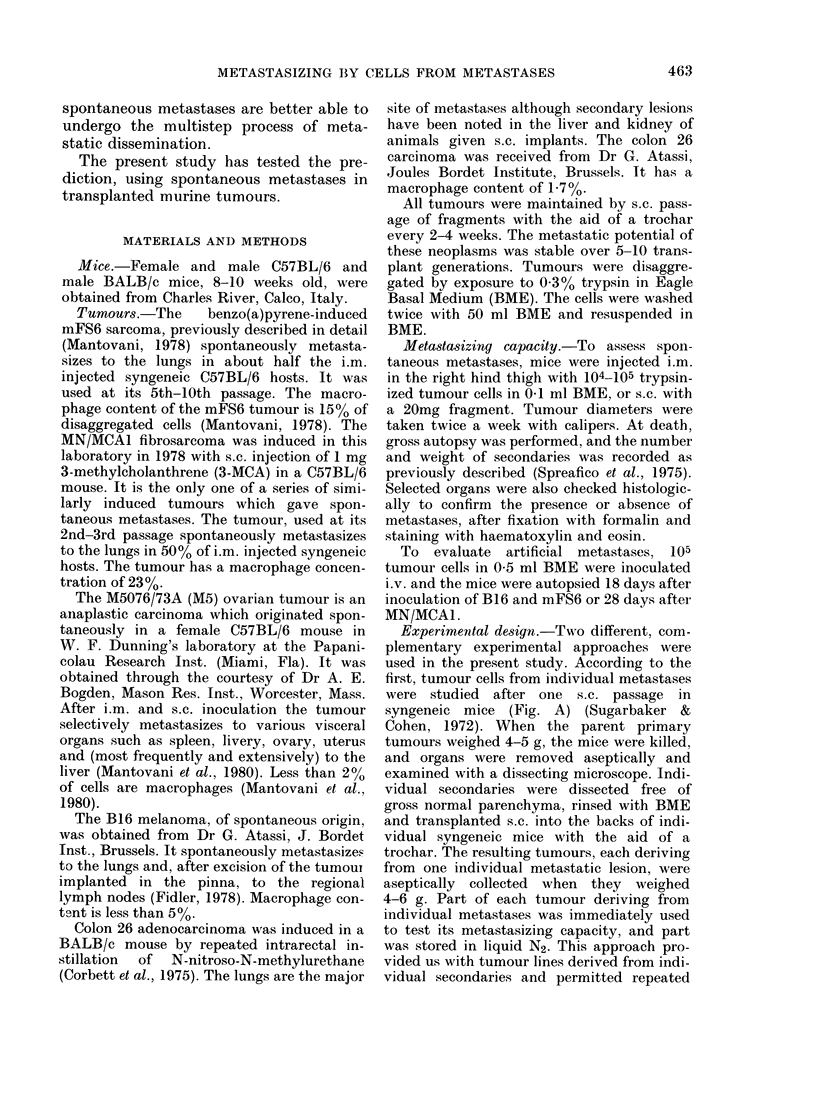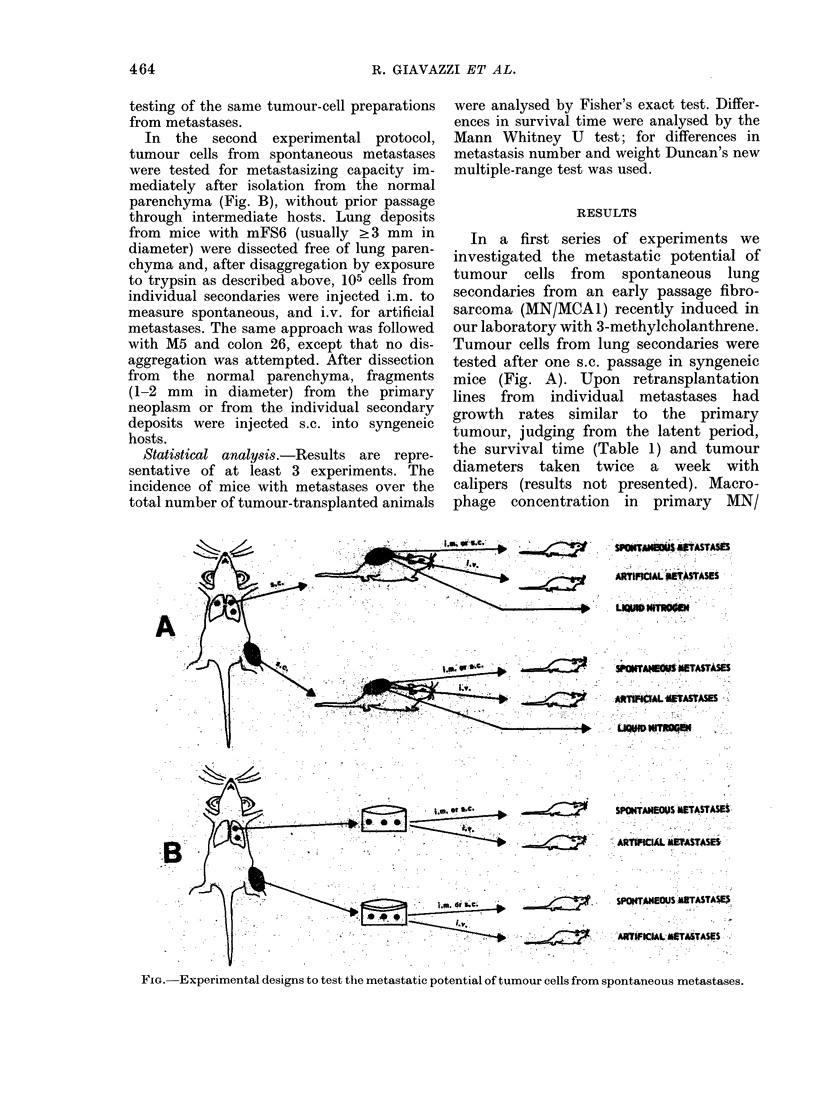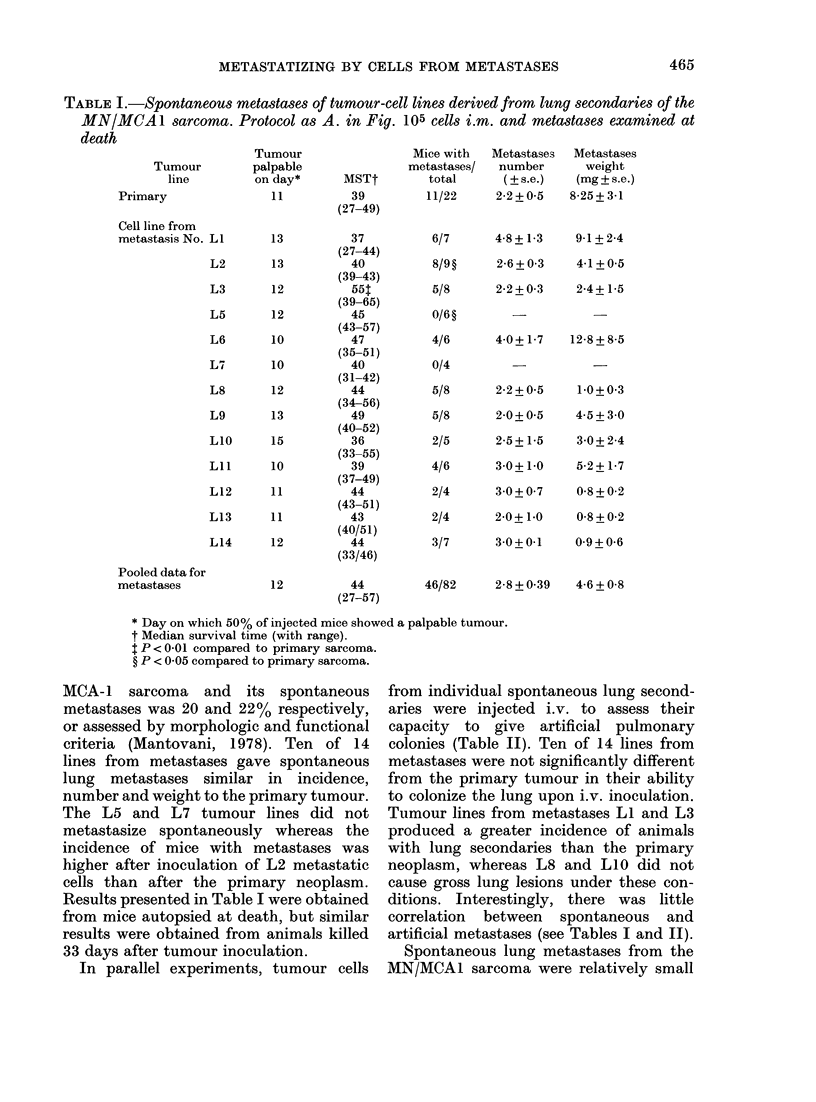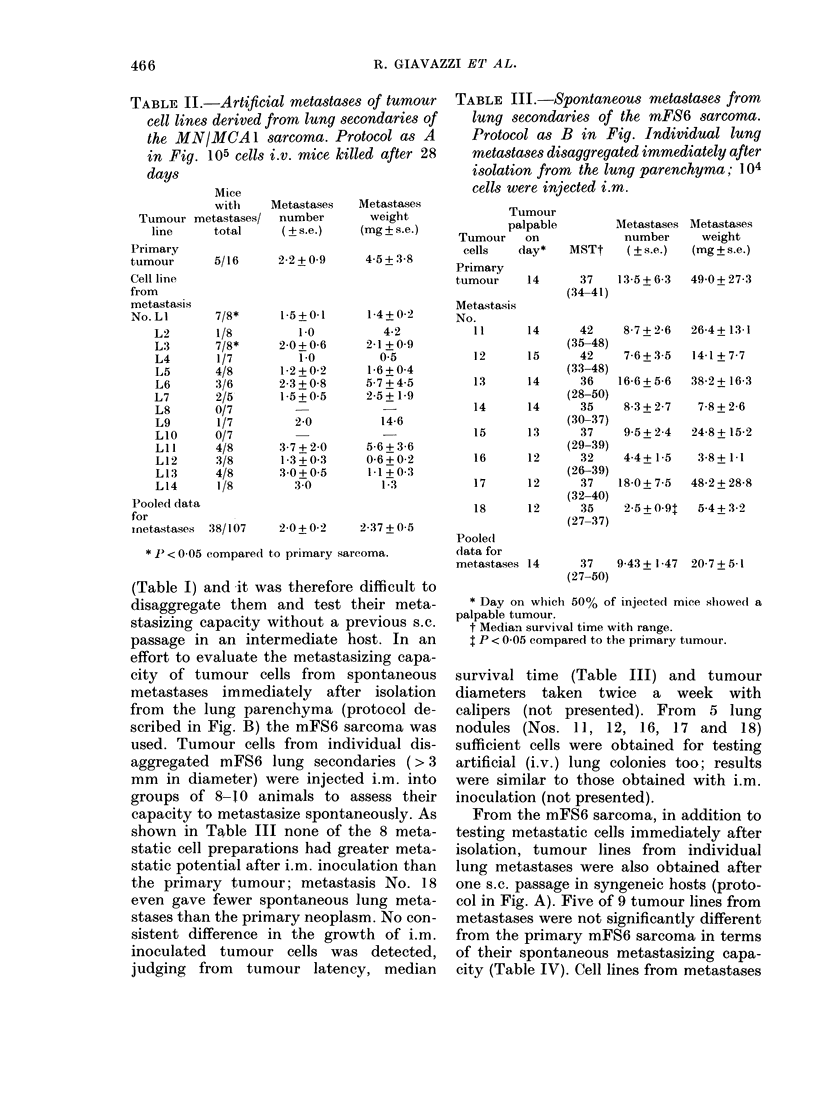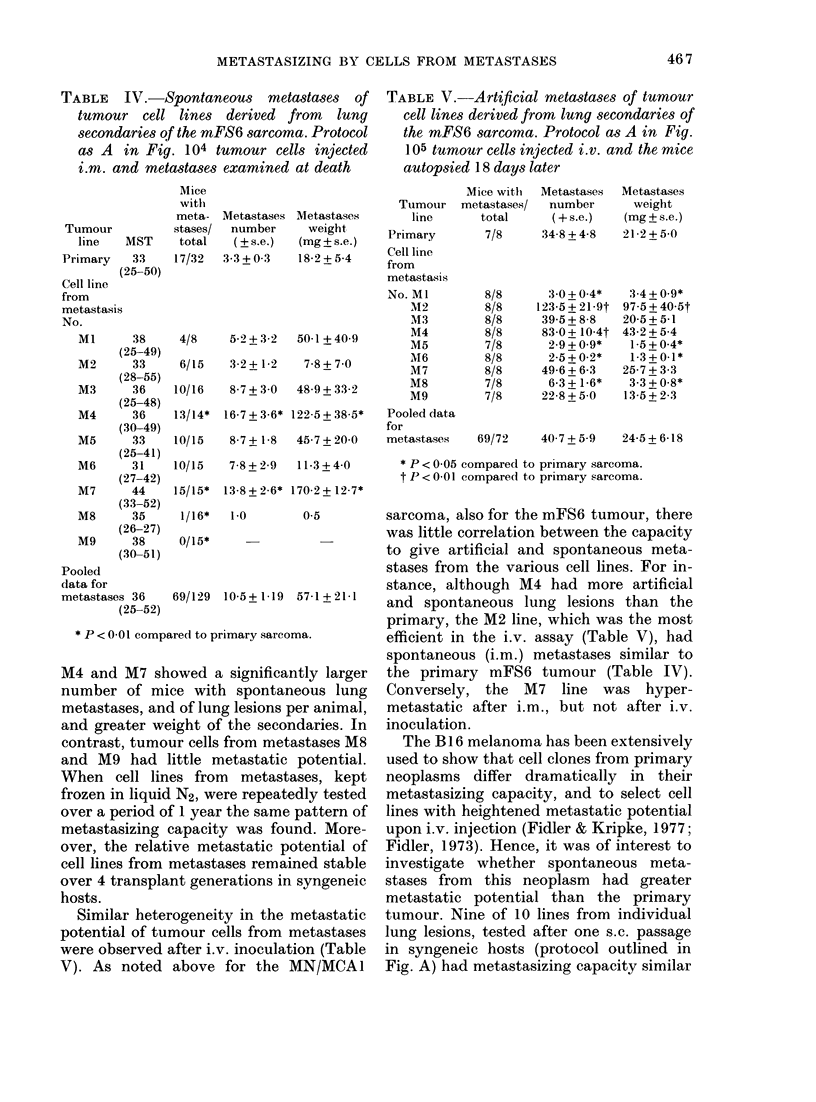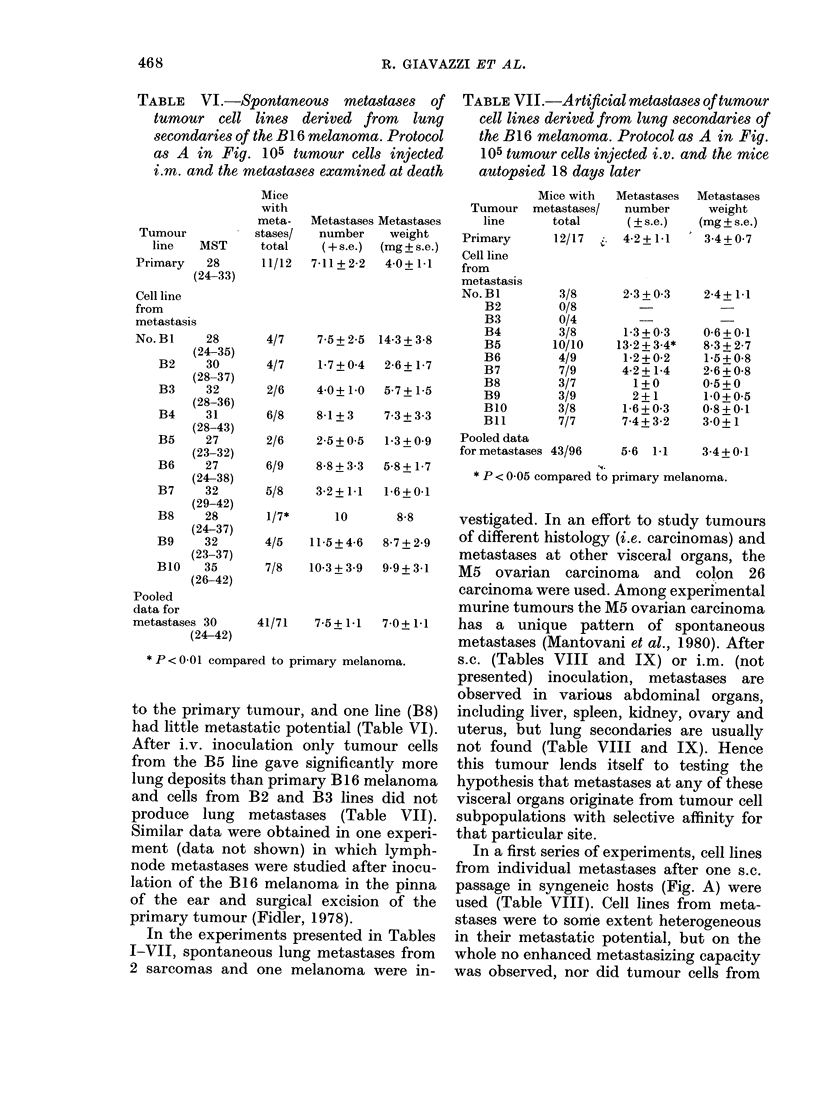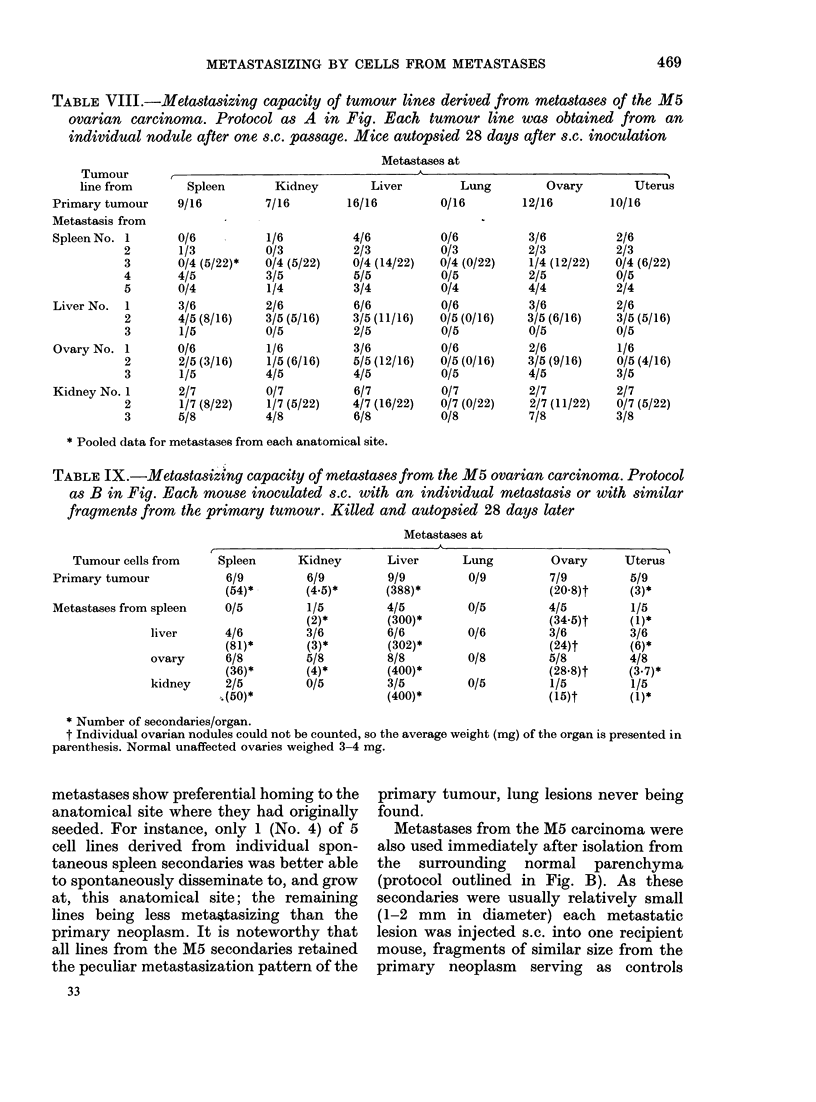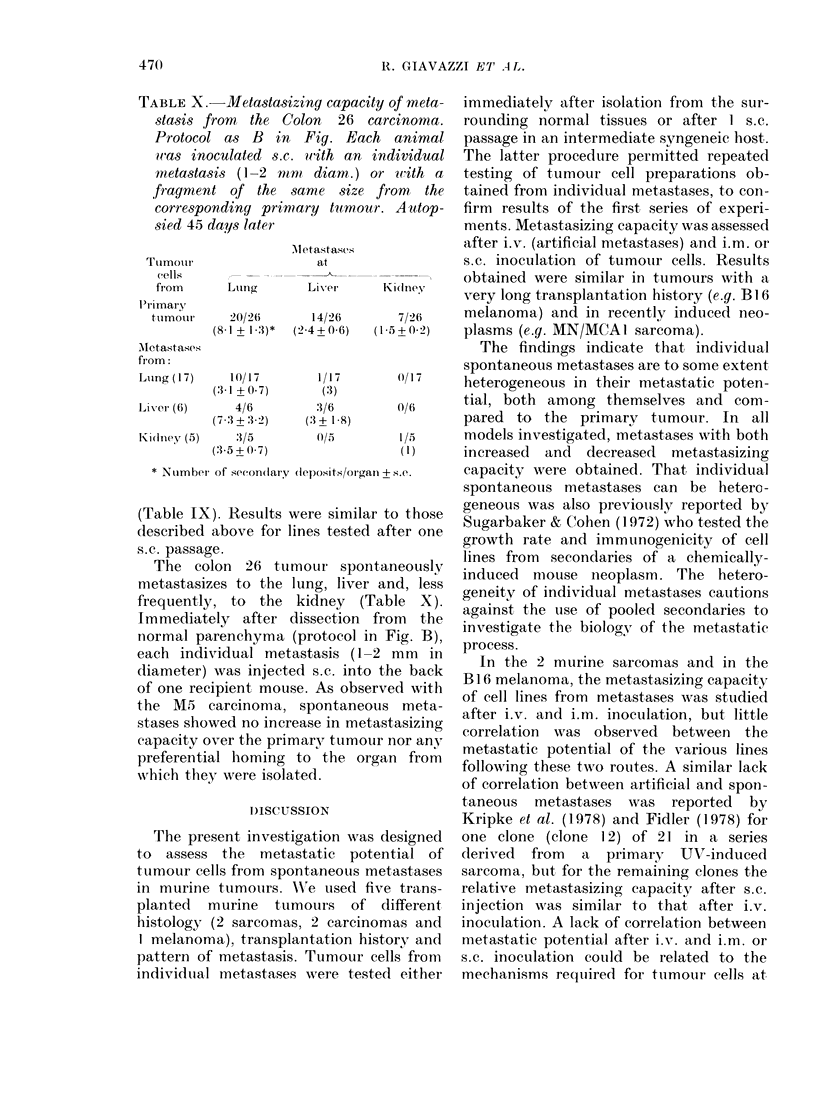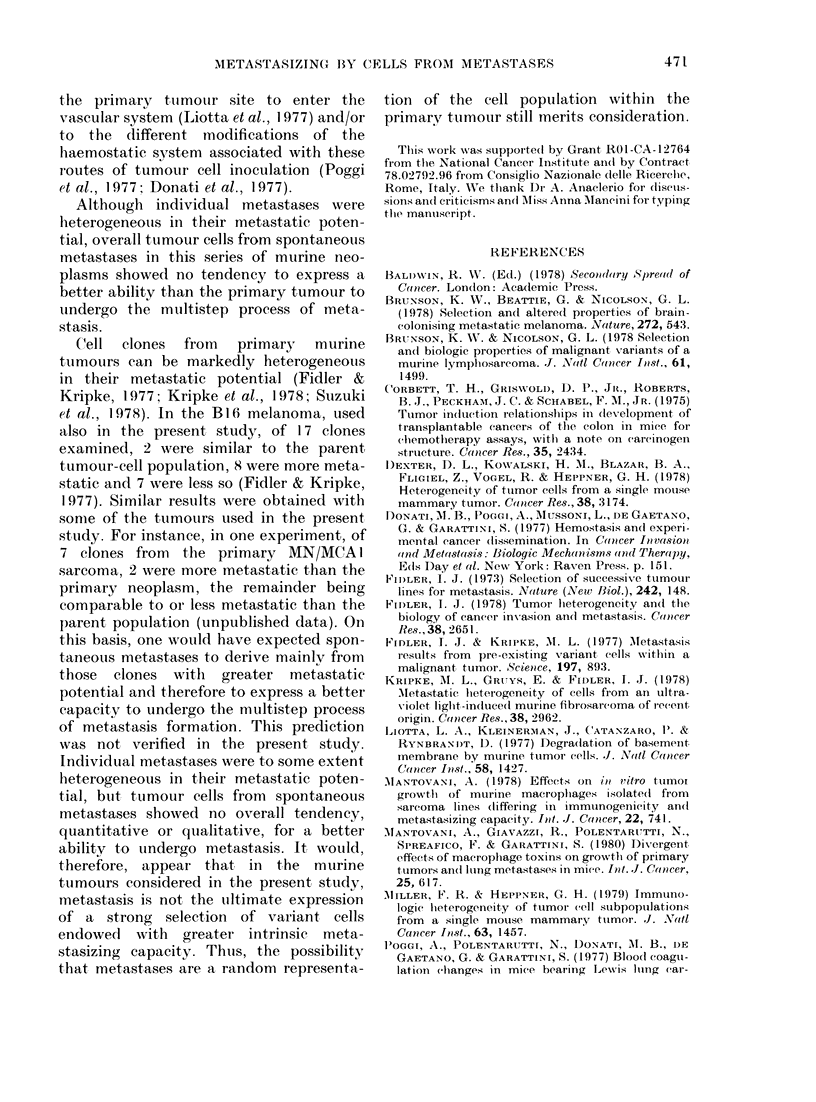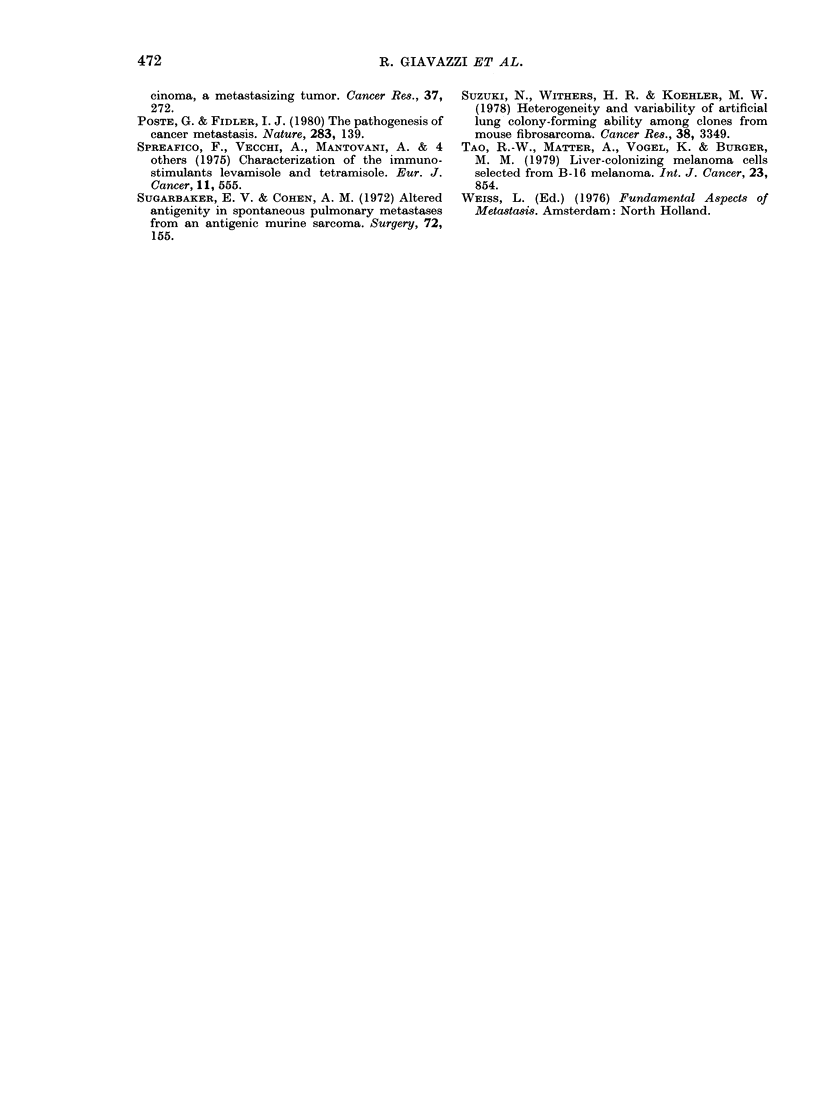# Metastasizing capacity of tumour cells from spontaneous metastases of transplanted murine tumours.

**DOI:** 10.1038/bjc.1980.259

**Published:** 1980-09

**Authors:** R. Giavazzi, G. Alessandri, F. Spreafico, S. Garattini, A. Mantovani

## Abstract

We investigated the metastasizing capacity of spontaneous lung metastases from the MN/MCA1 and mFS6 sarcoma, the B16 melanoma and colon 26 carcinoma. Spontaneous metastases at other visceral organs (liver, spleen, kidney, ovary, uterus) from the M5076/73A (M5) ovarian carcinoma and colon 26 carcinoma were also studied. Tumour cells from individual spontaneous metastases were used immediately after isolation from the normal parenchyma (mFS6, M5 and colon 26) and/or after 1 s.c. passage in syngeneic mice (MN/MCA1, mFS6, B16 and M5). Spontaneous metastases were examined for all tumours and their secondaries after i.m. or s.c. inoculation of tumour cells; artificial lung colonies were measured after i.v. injection only of cells from the primary mFS6 and MN/MCA1 and B16 or their spontaneous metastases. Individual spontaneous metastases were to some extent heterogeneous in their metastatic potential, a minority of the secondaries having greater or lesser metastatic capacity than the appropriate primary. Overall, tumour cells from spontaneous metastases did not show greater metastasizing capacity than primary neoplasms, nor was there evidence that metastases from specific organs (e.g. spleen and kidney) tended to home to the specific anatomical sites from which they were originally isolated. These observations in a series of murine tumours of different histology, transplantation history and pattern of metastasis, do not support the hypothesis that metastases are the ultimate expression of strong selection of variant cells with greater intrinsic metastatic potential, pre-existing within the primary tumour.


*Br. J. Cancer* (1980) **42**, 462

### METASTASIZING CAPACITY OF TUMOUR CELLS FROM SPONTANEOUS METASTASES OF TRANSPLANTED MURINE TUMOURS

R. GIAVAZZI, G. ALESSANDRI, F. SPREAFICO, S. GARATTINI AND A. MANTOVANI*

*From the Instituto di Ricerche Farmacologiche "Mario Negri", Via Eritrea, 62-20157 Milano, Italy*

Received 18 April 1980 Accepted 13 June 1980

**Summary.—We investigated the metastasizing capacity of spontaneous lung metastases from the MN/MCA1 and mFS6 sarcomas, the B16 melanoma and colon 26 carcinoma. Spontaneous metastases at other visceral organs (liver, spleen, kidney, ovary, uterus) from the M5076/73A (M5) ovarian carcinoma and colon 26 carcinoma were also studied. Tumour cells from individual spontaneous metastases were used immediately after isolation from the normal parenchyma (mFS6, M5 and colon 26) and/or after 1 s.c. passage in syngeneic mice (MN/MCA1, mFS6, B16 and M5). Spontaneous metastases were examined for all tumours and their secondaries after i.m. or s.c. inoculation of tumour cells; artificial lung colonies were measured after i.v. injection only of cells from the primary mFS6 and MN/MCA1 and B16 or their spontaneous metastases.**

**Individual spontaneous metastases were to some extent heterogeneous in their metastatic potential, a minority of the secondaries having greater or lesser metastatic capacity than the appropriate primary. Overall, tumour cells from spontaneous metastases did not show greater metastasizing capacity than primary neoplasms, nor was there evidence that metastases from specific organs (e.g. spleen and kidney) tended to home to the specific anatomical sites from which they were originally isolated.**

**These observations in a series of murine tumours of different histology, transplantation history and pattern of metastasis, do not support the hypothesis that metastases are the ultimate expression of strong selection of variant cells with greater intrinsic metastatic potential, pre-existing within the primary tumour.**

METASTASIS is one of the crucial events in malignancy, but the mechanisms involved in the processes of cancer cell dissemination and metastasis still largely remain to be defined (Weiss, 1976; Fidler, 1978; Baldwin, 1978; Poste & Fidler, 1980). Tumour-cell clones from primary murine neoplasms can be markedly heterogeneous in several biological characteristics, including metastasizing capacity (Fidler & Kripke, 1977; Kripke *et al.*, 1978; Suzuki *et al.*, 1978; Dexter *et al.*, 1978; Miller & Heppner, 1979). Tumour lines have been selected which, upon i.v. inoculation, show enhanced metastatic capacity (Fidler, 1973) or which selectively seed at specific anatomical sites (Brunson & Nicolson, 1978; Brunson *et al.*, 1978; Tao *et al.*, 1979). Essentially on the basis of these findings, it has been proposed that metastases originate from variant cells with greater intrinsic metastasizing capacity, pre-existing within the primary neoplasm, and that metastasis is the ultimate expression of a strongly selective multistep process (Fidler, 1978; Kripke *et al.*, 1978; Poste & Fidler, 1980).

This hypothesis predicts that cells from

* To whom correspondence should be addressed.

spontaneous metastases are better able to undergo the multistep process of metastatic dissemination.

The present study has tested the prediction, using spontaneous metastases in transplanted murine tumours.

### MATERIALS AND METHODS

*Mice.*—Female and male C57BL/6 and male BALB/c mice, 8–10 weeks old, were obtained from Charles River, Calco, Italy.

*Tumours.*—The benzo(a)pyrene-induced mFS6 sarcoma, previously described in detail (Mantovani, 1978) spontaneously metastasizes to the lungs in about half the i.m. injected syngeneic C57BL/6 hosts. It was used at its 5th–10th passage. The macrophage content of the mFS6 tumour is 15% of disaggregated cells (Mantovani, 1978). The MN/MCA1 fibrosarcoma was induced in this laboratory in 1978 with s.c. injection of 1 mg 3-methylcholanthrene (3-MCA) in a C57BL/6 mouse. It is the only one of a series of similarly induced tumours which gave spontaneous metastases. The tumour, used at its 2nd–3rd passage spontaneously metastasizes to the lungs in 50% of i.m. injected syngeneic hosts. The tumour has a macrophage concentration of 23%.

The M5076/73A (M5) ovarian tumour is an anaplastic carcinoma which originated spontaneously in a female C57BL/6 mouse in W. F. Dunning's laboratory at the Papanicolau Research Inst. (Miami, Fla). It was obtained through the courtesy of Dr A. E. Bogden, Mason Res. Inst., Worcester, Mass. After i.m. and s.c. inoculation the tumour selectively metastasizes to various visceral organs such as spleen, livery, ovary, uterus and (most frequently and extensively) to the liver (Mantovani *et al.*, 1980). Less than 2% of cells are macrophages (Mantovani *et al.*, 1980).

The B16 melanoma, of spontaneous origin, was obtained from Dr G. Atassi, J. Bordet Inst., Brussels. It spontaneously metastasizes to the lungs and, after excision of the tumour implanted in the pinna, to the regional lymph nodes (Fidler, 1978). Macrophage content is less than 5%.

Colon 26 adenocarcinoma was induced in a BALB/c mouse by repeated intrarectal instillation of N-nitroso-N-methylurethane (Corbett *et al.*, 1975). The lungs are the major site of metastases although secondary lesions have been noted in the liver and kidney of animals given s.c. implants. The colon 26 carcinoma was received from Dr G. Atassi, Joules Bordet Institute, Brussels. It has a macrophage content of 1·7%.

All tumours were maintained by s.c. passage of fragments with the aid of a trochar every 2–4 weeks. The metastatic potential of these neoplasms was stable over 5–10 transplant generations. Tumours were disaggregated by exposure to 0·3% trypsin in Eagle Basal Medium (BME). The cells were washed twice with 50 ml BME and resuspended in BME.

*Metastasizing capacity.*—To assess spontaneous metastases, mice were injected i.m. in the right hind thigh with $10^4$–$10^5$ trypsinized tumour cells in 0·1 ml BME, or s.c. with a 20mg fragment. Tumour diameters were taken twice a week with calipers. At death, gross autopsy was performed, and the number and weight of secondaries was recorded as previously described (Spreafico *et al.*, 1975). Selected organs were also checked histologically to confirm the presence or absence of metastases, after fixation with formalin and staining with haematoxylin and eosin.

To evaluate artificial metastases, $10^5$ tumour cells in 0·5 ml BME were inoculated i.v. and the mice were autopsied 18 days after inoculation of B16 and mFS6 or 28 days after MN/MCA1.

*Experimental design.*—Two different, complementary experimental approaches were used in the present study. According to the first, tumour cells from individual metastases were studied after one s.c. passage in syngeneic mice (Fig. A) (Sugarbaker & Cohen, 1972). When the parent primary tumours weighed 4–5 g, the mice were killed, and organs were removed aseptically and examined with a dissecting microscope. Individual secondaries were dissected free of gross normal parenchyma, rinsed with BME and transplanted s.c. into the backs of individual syngeneic mice with the aid of a trochar. The resulting tumours, each deriving from one individual metastatic lesion, were aseptically collected when they weighed 4–6 g. Part of each tumour deriving from individual metastases was immediately used to test its metastasizing capacity, and part was stored in liquid $N_2$. This approach provided us with tumour lines derived from individual secondaries and permitted repeated testing of the same tumour-cell preparations from metastases.

In the second experimental protocol, tumour cells from spontaneous metastases were tested for metastasizing capacity immediately after isolation from the normal parenchyma (Fig. B), without prior passage through intermediate hosts. Lung deposits from mice with mFS6 (usually ≥3 mm in diameter) were dissected free of lung parenchyma and, after disaggregation by exposure to trypsin as described above, $10^5$ cells from individual secondaries were injected i.m. to measure spontaneous, and i.v. for artificial metastases. The same approach was followed with M5 and colon 26, except that no disaggregation was attempted. After dissection from the normal parenchyma, fragments (1–2 mm in diameter) from the primary neoplasm or from the individual secondary deposits were injected s.c. into syngeneic hosts.

*Statistical analysis.*—Results are representative of at least 3 experiments. The incidence of mice with metastases over the total number of tumour-transplanted animals were analysed by Fisher's exact test. Differences in survival time were analysed by the Mann Whitney U test; for differences in metastasis number and weight Duncan's new multiple-range test was used.

### RESULTS

In a first series of experiments we investigated the metastatic potential of tumour cells from spontaneous lung secondaries from an early passage fibrosarcoma (MN/MCA1) recently induced in our laboratory with 3-methylcholanthrene. Tumour cells from lung secondaries were tested after one s.c. passage in syngeneic mice (Fig. A). Upon retransplantation lines from individual metastases had growth rates similar to the primary tumour, judging from the latent period, the survival time (Table 1) and tumour diameters taken twice a week with calipers (results not presented). Macrophage concentration in primary MN/

FIG.—Experimental designs to test the metastatic potential of tumour cells from spontaneous metastases.

TABLE I.—*Spontaneous metastases of tumour-cell lines derived from lung secondaries of the MN/MCA1 sarcoma. Protocol as A. in Fig.* $10^5$ *cells i.m. and metastases examined at death*

| Tumour line | | Tumour palpable on day* | MST† | Mice with metastases/ total | Metastases number (±s.e.) | Metastases weight (mg±s.e.) |
|---|---|---|---|---|---|---|
| Primary | | 11 | 39 (27–49) | 11/22 | 2·2±0·5 | 8·25±3·1 |
| Cell line from metastasis No. | L1 | 13 | 37 (27–44) | 6/7 | 4·8±1·3 | 9·1±2·4 |
| | L2 | 13 | 40 (39–43) | 8/9§ | 2·6±0·3 | 4·1±0·5 |
| | L3 | 12 | 55‡ (39–65) | 5/8 | 2·2±0·3 | 2·4±1·5 |
| | L5 | 12 | 45 (43–57) | 0/6§ | — | — |
| | L6 | 10 | 47 (35–51) | 4/6 | 4·0±1·7 | 12·8±8·5 |
| | L7 | 10 | 40 (31–42) | 0/4 | — | — |
| | L8 | 12 | 44 (34–56) | 5/8 | 2·2±0·5 | 1·0±0·3 |
| | L9 | 13 | 49 (40–52) | 5/8 | 2·0±0·5 | 4·5±3·0 |
| | L10 | 15 | 36 (33–55) | 2/5 | 2·5±1·5 | 3·0±2·4 |
| | L11 | 10 | 39 (37–49) | 4/6 | 3·0±1·0 | 5·2±1·7 |
| | L12 | 11 | 44 (43–51) | 2/4 | 3·0±0·7 | 0·8±0·2 |
| | L13 | 11 | 43 (40/51) | 2/4 | 2·0±1·0 | 0·8±0·2 |
| | L14 | 12 | 44 (33/46) | 3/7 | 3·0±0·1 | 0·9±0·6 |
| Pooled data for metastases | | 12 | 44 (27–57) | 46/82 | 2·8±0·39 | 4·6±0·8 |

* Day on which 50% of injected mice showed a palpable tumour.
† Median survival time (with range).
‡ $P < 0{\cdot}01$ compared to primary sarcoma.
§ $P < 0{\cdot}05$ compared to primary sarcoma.

MCA-1 sarcoma and its spontaneous metastases was 20 and 22% respectively, or assessed by morphologic and functional criteria (Mantovani, 1978). Ten of 14 lines from metastases gave spontaneous lung metastases similar in incidence, number and weight to the primary tumour. The L5 and L7 tumour lines did not metastasize spontaneously whereas the incidence of mice with metastases was higher after inoculation of L2 metastatic cells than after the primary neoplasm. Results presented in Table I were obtained from mice autopsied at death, but similar results were obtained from animals killed 33 days after tumour inoculation.

In parallel experiments, tumour cells from individual spontaneous lung secondaries were injected i.v. to assess their capacity to give artificial pulmonary colonies (Table II). Ten of 14 lines from metastases were not significantly different from the primary tumour in their ability to colonize the lung upon i.v. inoculation. Tumour lines from metastases L1 and L3 produced a greater incidence of animals with lung secondaries than the primary neoplasm, whereas L8 and L10 did not cause gross lung lesions under these conditions. Interestingly, there was little correlation between spontaneous and artificial metastases (see Tables I and II).

Spontaneous lung metastases from the MN/MCA1 sarcoma were relatively small

TABLE II.—*Artificial metastases of tumour cell lines derived from lung secondaries of the MN/MCA1 sarcoma. Protocol as A in Fig.* $10^5$ *cells i.v. mice killed after* 28 *days*

| Tumour line | Mice with metastases/ total | Metastases number (±s.e.) | Metastases weight (mg±s.e.) |
|---|---|---|---|
| Primary tumour | 5/16 | 2·2±0·9 | 4·5±3·8 |
| Cell line from metastasis | | | |
| No. L1 | 7/8* | 1·5±0·1 | 1·4±0·2 |
| L2 | 1/8 | 1·0 | 4·2 |
| L3 | 7/8* | 2·0±0·6 | 2·1±0·9 |
| L4 | 1/7 | 1·0 | 0·5 |
| L5 | 4/8 | 1·2±0·2 | 1·6±0·4 |
| L6 | 3/6 | 2·3±0·8 | 5·7±4·5 |
| L7 | 2/5 | 1·5±0·5 | 2·5±1·9 |
| L8 | 0/7 | — | — |
| L9 | 1/7 | 2·0 | 14·6 |
| L10 | 0/7 | — | — |
| L11 | 4/8 | 3·7±2·0 | 5·6±3·6 |
| L12 | 3/8 | 1·3±0·3 | 0·6±0·2 |
| L13 | 4/8 | 3·0±0·5 | 1·1±0·3 |
| L14 | 1/8 | 3·0 | 1·3 |
| Pooled data for metastases | 38/107 | 2·0±0·2 | 2·37±0·5 |

* $P < 0·05$ compared to primary sarcoma.

TABLE III.—*Spontaneous metastases from lung secondaries of the mFS6 sarcoma. Protocol as B in Fig. Individual lung metastases disaggregated immediately after isolation from the lung parenchyma;* $10^4$ *cells were injected i.m.*

| Tumour cells | Tumour palpable on day* | MST† | Metastases number (±s.e.) | Metastases weight (mg±s.e.) |
|---|---|---|---|---|
| Primary tumour | 14 | 37 (34–41) | 13·5±6·3 | 49·0±27·3 |
| Metastasis No. | | | | |
| 11 | 14 | 42 (35–48) | 8·7±2·6 | 26·4±13·1 |
| 12 | 15 | 42 (33–48) | 7·6±3·5 | 14·1±7·7 |
| 13 | 14 | 36 (28–50) | 16·6±5·6 | 38·2±16·3 |
| 14 | 14 | 35 (30–37) | 8·3±2·7 | 7·8±2·6 |
| 15 | 13 | 37 (29–39) | 9·5±2·4 | 24·8±15·2 |
| 16 | 12 | 32 (26–39) | 4·4±1·5 | 3·8±1·1 |
| 17 | 12 | 37 (32–40) | 18·0±7·5 | 48·2±28·8 |
| 18 | 12 | 35 (27–37) | 2·5±0·9‡ | 5·4±3·2 |
| Pooled data for metastases | 14 | 37 (27–50) | 9·43±1·47 | 20·7±5·1 |

* Day on which 50% of injected mice showed a palpable tumour.
† Median survival time with range.
‡ $P < 0·05$ compared to the primary tumour.

(Table I) and it was therefore difficult to disaggregate them and test their metastasizing capacity without a previous s.c. passage in an intermediate host. In an effort to evaluate the metastasizing capacity of tumour cells from spontaneous metastases immediately after isolation from the lung parenchyma (protocol described in Fig. B) the mFS6 sarcoma was used. Tumour cells from individual disaggregated mFS6 lung secondaries (>3 mm in diameter) were injected i.m. into groups of 8–10 animals to assess their capacity to metastasize spontaneously. As shown in Table III none of the 8 metastatic cell preparations had greater metastatic potential after i.m. inoculation than the primary tumour; metastasis No. 18 even gave fewer spontaneous lung metastases than the primary neoplasm. No consistent difference in the growth of i.m. inoculated tumour cells was detected, judging from tumour latency, median survival time (Table III) and tumour diameters taken twice a week with calipers (not presented). From 5 lung nodules (Nos. 11, 12, 16, 17 and 18) sufficient cells were obtained for testing artificial (i.v.) lung colonies too; results were similar to those obtained with i.m. inoculation (not presented).

From the mFS6 sarcoma, in addition to testing metastatic cells immediately after isolation, tumour lines from individual lung metastases were also obtained after one s.c. passage in syngeneic hosts (protocol in Fig. A). Five of 9 tumour lines from metastases were not significantly different from the primary mFS6 sarcoma in terms of their spontaneous metastasizing capacity (Table IV). Cell lines from metastases

TABLE IV.—*Spontaneous metastases of tumour cell lines derived from lung secondaries of the mFS6 sarcoma. Protocol as A in Fig.* $10^4$ *tumour cells injected i.m. and metastases examined at death*

| Tumour line | MST | Mice with metastases/ total | Metastases number (±s.e.) | Metastases weight (mg±s.e.) |
|---|---|---|---|---|
| Primary | 33 (25–50) | 17/32 | 3·3±0·3 | 18·2±5·4 |
| Cell line from metastasis No. | | | | |
| M1 | 38 (25–49) | 4/8 | 5·2±3·2 | 50·1±40·9 |
| M2 | 33 (28–55) | 6/15 | 3·2±1·2 | 7·8±7·0 |
| M3 | 36 (25–48) | 10/16 | 8·7±3·0 | 48·9±33·2 |
| M4 | 36 (30–49) | 13/14* | 16·7±3·6* | 122·5±38·5* |
| M5 | 33 (25–41) | 10/15 | 8·7±1·8 | 45·7±20·0 |
| M6 | 31 (27–42) | 10/15 | 7·8±2·9 | 11·3±4·0 |
| M7 | 44 (33–52) | 15/15* | 13·8±2·6* | 170·2±12·7* |
| M8 | 35 (26–27) | 1/16* | 1·0 | 0·5 |
| M9 | 38 (30–51) | 0/15* | — | — |
| Pooled data for metastases | 36 (25–52) | 69/129 | 10·5±1·19 | 57·1±21·1 |

* $P < 0{\cdot}01$ compared to primary sarcoma.

M4 and M7 showed a significantly larger number of mice with spontaneous lung metastases, and of lung lesions per animal, and greater weight of the secondaries. In contrast, tumour cells from metastases M8 and M9 had little metastatic potential. When cell lines from metastases, kept frozen in liquid $N_2$, were repeatedly tested over a period of 1 year the same pattern of metastasizing capacity was found. Moreover, the relative metastatic potential of cell lines from metastases remained stable over 4 transplant generations in syngeneic hosts.

Similar heterogeneity in the metastatic potential of tumour cells from metastases were observed after i.v. inoculation (Table V). As noted above for the MN/MCA1

TABLE V.—*Artificial metastases of tumour cell lines derived from lung secondaries of the mFS6 sarcoma. Protocol as A in Fig.* $10^5$ *tumour cells injected i.v. and the mice autopsied* 18 *days later*

| Tumour line | Mice with metastases/ total | Metastases number (+s.e.) | Metastases weight (mg±s.e.) |
|---|---|---|---|
| Primary | 7/8 | 34·8±4·8 | 21·2±5·0 |
| Cell line from metastasis | | | |
| No. M1 | 8/8 | 3·0±0·4* | 3·4±0·9* |
| M2 | 8/8 | 123·5±21·9† | 97·5±40·5† |
| M3 | 8/8 | 39·5±8·8 | 20·5±5·1 |
| M4 | 8/8 | 83·0±10·4† | 43·2±5·4 |
| M5 | 7/8 | 2·9±0·9* | 1·5±0·4* |
| M6 | 8/8 | 2·5±0·2* | 1·3±0·1* |
| M7 | 8/8 | 49·6±6·3 | 25·7±3·3 |
| M8 | 7/8 | 6·3±1·6* | 3·3±0·8* |
| M9 | 7/8 | 22·8±5·0 | 13·5±2·3 |
| Pooled data for metastases | 69/72 | 40·7±5·9 | 24·5±6·18 |

* $P < 0{\cdot}05$ compared to primary sarcoma.
† $P < 0{\cdot}01$ compared to primary sarcoma.

sarcoma, also for the mFS6 tumour, there was little correlation between the capacity to give artificial and spontaneous metastases from the various cell lines. For instance, although M4 had more artificial and spontaneous lung lesions than the primary, the M2 line, which was the most efficient in the i.v. assay (Table V), had spontaneous (i.m.) metastases similar to the primary mFS6 tumour (Table IV). Conversely, the M7 line was hypermetastatic after i.m., but not after i.v. inoculation.

The B16 melanoma has been extensively used to show that cell clones from primary neoplasms differ dramatically in their metastasizing capacity, and to select cell lines with heightened metastatic potential upon i.v. injection (Fidler & Kripke, 1977; Fidler, 1973). Hence, it was of interest to investigate whether spontaneous metastases from this neoplasm had greater metastatic potential than the primary tumour. Nine of 10 lines from individual lung lesions, tested after one s.c. passage in syngeneic hosts (protocol outlined in Fig. A) had metastasizing capacity similar

TABLE VI.—*Spontaneous metastases of tumour cell lines derived from lung secondaries of the B*16 *melanoma. Protocol as A in Fig.* $10^5$ *tumour cells injected i.m. and the metastases examined at death*

| Tumour line | MST | Mice with metastases/ total | Metastases number (+s.e.) | Metastases weight (mg ± s.e.) |
|---|---|---|---|---|
| Primary | 28 (24–33) | 11/12 | 7·11 ± 2·2 | 4·0 ± 1·1 |
| Cell line from metastasis | | | | |
| No. B1 | 28 (24–35) | 4/7 | 7·5 ± 2·5 | 14·3 ± 3·8 |
| B2 | 30 (28–37) | 4/7 | 1·7 ± 0·4 | 2·6 ± 1·7 |
| B3 | 32 (28–36) | 2/6 | 4·0 ± 1·0 | 5·7 ± 1·5 |
| B4 | 31 (28–43) | 6/8 | 8·1 ± 3 | 7·3 ± 3·3 |
| B5 | 27 (23–32) | 2/6 | 2·5 ± 0·5 | 1·3 ± 0·9 |
| B6 | 27 (24–38) | 6/9 | 8·8 ± 3·3 | 5·8 ± 1·7 |
| B7 | 32 (29–42) | 5/8 | 3·2 ± 1·1 | 1·6 ± 0·1 |
| B8 | 28 (24–37) | 1/7* | 10 | 8·8 |
| B9 | 32 (23–37) | 4/5 | 11·5 ± 4·6 | 8·7 ± 2·9 |
| B10 | 35 (26–42) | 7/8 | 10·3 ± 3·9 | 9·9 ± 3·1 |
| Pooled data for metastases | 30 (24–42) | 41/71 | 7·5 ± 1·1 | 7·0 ± 1·1 |

* $P < 0·01$ compared to primary melanoma.

TABLE VII.—*Artificial metastases of tumour cell lines derived from lung secondaries of the B*16 *melanoma. Protocol as A in Fig.* $10^5$ *tumour cells injected i.v. and the mice autopsied* 18 *days later*

| Tumour line | Mice with metastases/ total | Metastases number (± s.e.) | Metastases weight (mg ± s.e.) |
|---|---|---|---|
| Primary | 12/17 | 4·2 ± 1·1 | 3·4 ± 0·7 |
| Cell line from metastasis | | | |
| No. B1 | 3/8 | 2·3 ± 0·3 | 2·4 ± 1·1 |
| B2 | 0/8 | — | — |
| B3 | 0/4 | — | — |
| B4 | 3/8 | 1·3 ± 0·3 | 0·6 ± 0·1 |
| B5 | 10/10 | 13·2 ± 3·4* | 8·3 ± 2·7 |
| B6 | 4/9 | 1·2 ± 0·2 | 1·5 ± 0·8 |
| B7 | 7/9 | 4·2 ± 1·4 | 2·6 ± 0·8 |
| B8 | 3/7 | 1 ± 0 | 0·5 ± 0 |
| B9 | 3/9 | 2 ± 1 | 1·0 ± 0·5 |
| B10 | 3/8 | 1·6 ± 0·3 | 0·8 ± 0·1 |
| B11 | 7/7 | 7·4 ± 3·2 | 3·0 ± 1 |
| Pooled data for metastases | 43/96 | 5·6 1·1 | 3·4 ± 0·1 |

* $P < 0·05$ compared to primary melanoma.

to the primary tumour, and one line (B8) had little metastatic potential (Table VI). After i.v. inoculation only tumour cells from the B5 line gave significantly more lung deposits than primary B16 melanoma and cells from B2 and B3 lines did not produce lung metastases (Table VII). Similar data were obtained in one experiment (data not shown) in which lymph-node metastases were studied after inoculation of the B16 melanoma in the pinna of the ear and surgical excision of the primary tumour (Fidler, 1978).

In the experiments presented in Tables I–VII, spontaneous lung metastases from 2 sarcomas and one melanoma were investigated. In an effort to study tumours of different histology (*i.e.* carcinomas) and metastases at other visceral organs, the M5 ovarian carcinoma and colon 26 carcinoma were used. Among experimental murine tumours the M5 ovarian carcinoma has a unique pattern of spontaneous metastases (Mantovani *et al.*, 1980). After s.c. (Tables VIII and IX) or i.m. (not presented) inoculation, metastases are observed in various abdominal organs, including liver, spleen, kidney, ovary and uterus, but lung secondaries are usually not found (Table VIII and IX). Hence this tumour lends itself to testing the hypothesis that metastases at any of these visceral organs originate from tumour cell subpopulations with selective affinity for that particular site.

In a first series of experiments, cell lines from individual metastases after one s.c. passage in syngeneic hosts (Fig. A) were used (Table VIII). Cell lines from metastases were to some extent heterogeneous in their metastatic potential, but on the whole no enhanced metastasizing capacity was observed, nor did tumour cells from

TABLE VIII.—*Metastasizing capacity of tumour lines derived from metastases of the M5 ovarian carcinoma. Protocol as A in Fig. Each tumour line was obtained from an individual nodule after one s.c. passage. Mice autopsied 28 days after s.c. inoculation*

| Tumour line from | Metastases at | | | | | |
|---|---|---|---|---|---|---|
| | Spleen | Kidney | Liver | Lung | Ovary | Uterus |
| Primary tumour | 9/16 | 7/16 | 16/16 | 0/16 | 12/16 | 10/16 |
| Metastasis from | | | | | | |
| Spleen No. 1 | 0/6 | 1/6 | 4/6 | 0/6 | 3/6 | 2/6 |
| 2 | 1/3 | 0/3 | 2/3 | 0/3 | 2/3 | 2/3 |
| 3 | 0/4 (5/22)* | 0/4 (5/22) | 0/4 (14/22) | 0/4 (0/22) | 1/4 (12/22) | 0/4 (6/22) |
| 4 | 4/5 | 3/5 | 5/5 | 0/5 | 2/5 | 0/5 |
| 5 | 0/4 | 1/4 | 3/4 | 0/4 | 4/4 | 2/4 |
| Liver No. 1 | 3/6 | 2/6 | 6/6 | 0/6 | 3/6 | 2/6 |
| 2 | 4/5 (8/16) | 3/5 (5/16) | 3/5 (11/16) | 0/5 (0/16) | 3/5 (6/16) | 3/5 (5/16) |
| 3 | 1/5 | 0/5 | 2/5 | 0/5 | 0/5 | 0/5 |
| Ovary No. 1 | 0/6 | 1/6 | 3/6 | 0/6 | 2/6 | 1/6 |
| 2 | 2/5 (3/16) | 1/5 (6/16) | 5/5 (12/16) | 0/5 (0/16) | 3/5 (9/16) | 0/5 (4/16) |
| 3 | 1/5 | 4/5 | 4/5 | 0/5 | 4/5 | 3/5 |
| Kidney No. 1 | 2/7 | 0/7 | 6/7 | 0/7 | 2/7 | 2/7 |
| 2 | 1/7 (8/22) | 1/7 (5/22) | 4/7 (16/22) | 0/7 (0/22) | 2/7 (11/22) | 0/7 (5/22) |
| 3 | 5/8 | 4/8 | 6/8 | 0/8 | 7/8 | 3/8 |

* Pooled data for metastases from each anatomical site.

TABLE IX.—*Metastasizing capacity of metastases from the M5 ovarian carcinoma. Protocol as B in Fig. Each mouse inoculated s.c. with an individual metastasis or with similar fragments from the primary tumour. Killed and autopsied 28 days later*

| Tumour cells from | Metastases at | | | | | |
|---|---|---|---|---|---|---|
| | Spleen | Kidney | Liver | Lung | Ovary | Uterus |
| Primary tumour | 6/9 (54)* | 6/9 (4·5)* | 9/9 (388)* | 0/9 | 7/9 (20·8)† | 5/9 (3)* |
| Metastases from spleen | 0/5 | 1/5 (2)* | 4/5 (300)* | 0/5 | 4/5 (34·5)† | 1/5 (1)* |
| liver | 4/6 (81)* | 3/6 (3)* | 6/6 (302)* | 0/6 | 3/6 (24)† | 3/6 (6)* |
| ovary | 6/8 (36)* | 5/8 (4)* | 8/8 (400)* | 0/8 | 5/8 (28·8)† | 4/8 (3·7)* |
| kidney | 2/5 (50)* | 0/5 | 3/5 (400)* | 0/5 | 1/5 (15)† | 1/5 (1)* |

* Number of secondaries/organ.
† Individual ovarian nodules could not be counted, so the average weight (mg) of the organ is presented in parenthesis. Normal unaffected ovaries weighed 3–4 mg.

metastases show preferential homing to the anatomical site where they had originally seeded. For instance, only 1 (No. 4) of 5 cell lines derived from individual spontaneous spleen secondaries was better able to spontaneously disseminate to, and grow at, this anatomical site; the remaining lines being less metastasizing than the primary neoplasm. It is noteworthy that all lines from the M5 secondaries retained the peculiar metastasization pattern of the primary tumour, lung lesions never being found.

Metastases from the M5 carcinoma were also used immediately after isolation from the surrounding normal parenchyma (protocol outlined in Fig. B). As these secondaries were usually relatively small (1–2 mm in diameter) each metastatic lesion was injected s.c. into one recipient mouse, fragments of similar size from the primary neoplasm serving as controls

TABLE X.—*Metastasizing capacity of metastasis from the Colon* 26 *carcinoma. Protocol as B in Fig. Each animal was inoculated s.c. with an individual metastasis* (1–2 *mm diam.*) *or with a fragment of the same size from the corresponding primary tumour. Autopsied* 45 *days later*

| Tumour cells from | Metastases at Lung | Liver | Kidney |
|---|---|---|---|
| Primary tumour | 20/26 (8·1 ± 1·3)* | 14/26 (2·4 ± 0·6) | 7/26 (1·5 ± 0·2) |
| Metastases from: | | | |
| Lung (17) | 10/17 (3·1 ± 0·7) | 1/17 (3) | 0/17 |
| Liver (6) | 4/6 (7·3 ± 3·2) | 3/6 (3 ± 1·8) | 0/6 |
| Kidney (5) | 3/5 (3·5 ± 0·7) | 0/5 | 1/5 (1) |

* Number of secondary deposits/organ ± s.e.

(Table IX). Results were similar to those described above for lines tested after one s.c. passage.

The colon 26 tumour spontaneously metastasizes to the lung, liver and, less frequently, to the kidney (Table X). Immediately after dissection from the normal parenchyma (protocol in Fig. B), each individual metastasis (1–2 mm in diameter) was injected s.c. into the back of one recipient mouse. As observed with the M5 carcinoma, spontaneous metastases showed no increase in metastasizing capacity over the primary tumour nor any preferential homing to the organ from which they were isolated.

### DISCUSSION

The present investigation was designed to assess the metastatic potential of tumour cells from spontaneous metastases in murine tumours. We used five transplanted murine tumours of different histology (2 sarcomas, 2 carcinomas and 1 melanoma), transplantation history and pattern of metastasis. Tumour cells from individual metastases were tested either immediately after isolation from the surrounding normal tissues or after 1 s.c. passage in an intermediate syngeneic host. The latter procedure permitted repeated testing of tumour cell preparations obtained from individual metastases, to confirm results of the first series of experiments. Metastasizing capacity was assessed after i.v. (artificial metastases) and i.m. or s.c. inoculation of tumour cells. Results obtained were similar in tumours with a very long transplantation history (*e.g.* B16 melanoma) and in recently induced neoplasms (*e.g.* MN/MCA1 sarcoma).

The findings indicate that individual spontaneous metastases are to some extent heterogeneous in their metastatic potential, both among themselves and compared to the primary tumour. In all models investigated, metastases with both increased and decreased metastasizing capacity were obtained. That individual spontaneous metastases can be heterogeneous was also previously reported by Sugarbaker & Cohen (1972) who tested the growth rate and immunogenicity of cell lines from secondaries of a chemically-induced mouse neoplasm. The heterogeneity of individual metastases cautions against the use of pooled secondaries to investigate the biology of the metastatic process.

In the 2 murine sarcomas and in the B16 melanoma, the metastasizing capacity of cell lines from metastases was studied after i.v. and i.m. inoculation, but little correlation was observed between the metastatic potential of the various lines following these two routes. A similar lack of correlation between artificial and spontaneous metastases was reported by Kripke *et al.* (1978) and Fidler (1978) for one clone (clone 12) of 21 in a series derived from a primary UV-induced sarcoma, but for the remaining clones the relative metastasizing capacity after s.c. injection was similar to that after i.v. inoculation. A lack of correlation between metastatic potential after i.v. and i.m. or s.c. inoculation could be related to the mechanisms required for tumour cells at the primary tumour site to enter the vascular system (Liotta *et al.*, 1977) and/or to the different modifications of the haemostatic system associated with these routes of tumour cell inoculation (Poggi *et al.*, 1977; Donati *et al.*, 1977).

Although individual metastases were heterogeneous in their metastatic potential, overall tumour cells from spontaneous metastases in this series of murine neoplasms showed no tendency to express a better ability than the primary tumour to undergo the multistep process of metastasis.

Cell clones from primary murine tumours can be markedly heterogeneous in their metastatic potential (Fidler & Kripke, 1977; Kripke *et al.*, 1978; Suzuki *et al.*, 1978). In the B16 melanoma, used also in the present study, of 17 clones examined, 2 were similar to the parent tumour-cell population, 8 were more metastatic and 7 were less so (Fidler & Kripke, 1977). Similar results were obtained with some of the tumours used in the present study. For instance, in one experiment, of 7 clones from the primary MN/MCA1 sarcoma, 2 were more metastatic than the primary neoplasm, the remainder being comparable to or less metastatic than the parent population (unpublished data). On this basis, one would have expected spontaneous metastases to derive mainly from those clones with greater metastatic potential and therefore to express a better capacity to undergo the multistep process of metastasis formation. This prediction was not verified in the present study. Individual metastases were to some extent heterogeneous in their metastatic potential, but tumour cells from spontaneous metastases showed no overall tendency, quantitative or qualitative, for a better ability to undergo metastasis. It would, therefore, appear that in the murine tumours considered in the present study, metastasis is not the ultimate expression of a strong selection of variant cells endowed with greater intrinsic metastasizing capacity. Thus, the possibility that metastases are a random representation of the cell population within the primary tumour still merits consideration.

This work was supported by Grant R01-CA-12764 from the National Cancer Institute and by Contract 78.02792.96 from Consiglio Nazionale delle Ricerche, Rome, Italy. We thank Dr A. Anaclerio for discussions and criticisms and Miss Anna Mancini for typing the manuscript.

### REFERENCES

BALDWIN, R. W. (Ed.) (1978) *Secondary Spread of Cancer*. London: Academic Press.

BRUNSON, K. W., BEATTIE, G. & NICOLSON, G. L. (1978) Selection and altered properties of brain-colonising metastatic melanoma. *Nature*, **272,** 543.

BRUNSON, K. W. & NICOLSON, G. L. (1978 Selection and biologic properties of malignant variants of a murine lymphosarcoma. *J. Natl Cancer Inst.*, **61,** 1499.

CORBETT, T. H., GRISWOLD, D. P., JR., ROBERTS, B. J., PECKHAM, J. C. & SCHABEL, F. M., JR. (1975) Tumor induction relationships in development of transplantable cancers of the colon in mice for chemotherapy assays, with a note on carcinogen structure. *Cancer Res.*, **35,** 2434.

DEXTER, D. L., KOWALSKI, H. M., BLAZAR, B. A., FLIGIEL, Z., VOGEL, R. & HEPPNER, G. H. (1978) Heterogeneity of tumor cells from a single mouse mammary tumor. *Cancer Res.*, **38,** 3174.

DONATI, M. B., POGGI, A., MUSSONI, L., DE GAETANO, G. & GARATTINI, S. (1977) Hemostasis and experimental cancer dissemination. In *Cancer Invasion and Metastasis: Biologic Mechanisms and Therapy*, Eds Day *et al.* New York: Raven Press. p. 151.

FIDLER, I. J. (1973) Selection of successive tumour lines for metastasis. *Nature* (*New Biol.*), **242,** 148.

FIDLER, I. J. (1978) Tumor heterogeneity and the biology of cancer invasion and metastasis. *Cancer Res.*, **38,** 2651.

FIDLER, I. J. & KRIPKE, M. L. (1977) Metastasis results from pre-existing variant cells within a malignant tumor. *Science*, **197,** 893.

KRIPKE, M. L., GRUYS, E. & FIDLER, I. J. (1978) Metastatic heterogeneity of cells from an ultraviolet light-induced murine fibrosarcoma of recent origin. *Cancer Res.*, **38,** 2962.

LIOTTA, L. A., KLEINERMAN, J., CATANZARO, P. & RYNBRANDT, D. (1977) Degradation of basement membrane by murine tumor cells. *J. Natl Cancer Cancer Inst.*, **58,** 1427.

MANTOVANI, A. (1978) Effects on *in vitro* tumor growth of murine macrophages isolated from sarcoma lines differing in immunogenicity and metastasizing capacity. *Int. J. Cancer*, **22,** 741.

MANTOVANI, A., GIAVAZZI, R., POLENTARUTTI, N., SPREAFICO, F. & GARATTINI, S. (1980) Divergent effects of macrophage toxins on growth of primary tumors and lung metastases in mice. *Int. J. Cancer*, **25,** 617.

MILLER, F. R. & HEPPNER, G. H. (1979) Immunologic heterogeneity of tumor cell subpopulations from a single mouse mammary tumor. *J. Natl Cancer Inst.*, **63,** 1457.

POGGI, A., POLENTARUTTI, N., DONATI, M. B., DE GAETANO, G. & GARATTINI, S. (1977) Blood coagulation changes in mice bearing Lewis lung carcinoma, a metastasizing tumor. *Cancer Res.*, **37**, 272.

POSTE, G. & FIDLER, I. J. (1980) The pathogenesis of cancer metastasis. *Nature*, **283**, 139.

SPREAFICO, F., VECCHI, A., MANTOVANI, A. & 4 others (1975) Characterization of the immunostimulants levamisole and tetramisole. *Eur. J. Cancer*, **11**, 555.

SUGARBAKER, E. V. & COHEN, A. M. (1972) Altered antigenity in spontaneous pulmonary metastases from an antigenic murine sarcoma. *Surgery*, **72**, 155.

SUZUKI, N., WITHERS, H. R. & KOEHLER, M. W. (1978) Heterogeneity and variability of artificial lung colony-forming ability among clones from mouse fibrosarcoma. *Cancer Res.*, **38**, 3349.

TAO, R.-W., MATTER, A., VOGEL, K. & BURGER, M. M. (1979) Liver-colonizing melanoma cells selected from B-16 melanoma. *Int. J. Cancer*, **23**, 854.

WEISS, L. (Ed.) (1976) *Fundamental Aspects of Metastasis*. Amsterdam: North Holland.